# Federated Learning Architecture for 3D Breast Cancer Image Classification

**DOI:** 10.3390/cancers17213450

**Published:** 2025-10-28

**Authors:** Amel Ali Alhussan, Wiem Nhidi, Imen Filali, Faten Benhmida, Ridha Ejbali

**Affiliations:** 1 Department of Computer Sciences, College of Computer and Information Sciences, Princess Nourah bint Abdulrahman University, Riyadh 84428, Saudi Arabia; aaalhussan@pnu.edu.sa (A.A.A.); imfilali@pnu.edu.sa (I.F.); fmbenhmida@pnu.edu.sa (F.B.); 2 Research Team on Intelligent Machines, National School of Engineers of Gabès, Zrig Eddakhlania 6029, Tunisia; wiem.nhidi@fsg.rnu.tn

**Keywords:** breast cancer, federate learning, detection, 3D mammography images

## Abstract

**Simple Summary:**

Breast cancer is one of the most common and deadly diseases affecting women. Detecting it early can save many lives, but developing accurate computer systems for diagnosis usually requires sharing large amounts of patient data, which raises privacy concerns. In this study, we introduce a new method that allows hospitals to work together to improve breast cancer detection without sharing any sensitive data. Instead of sending patient images to a central location, each hospital trains its own model locally and shares only the learned information. These updates are then combined to create a stronger, global model. Our approach focuses on advanced three-dimensional breast images, which provide more detailed information for diagnosis. This work could help medical institutions collaborate securely and develop powerful, privacy-preserving tools to improve early detection and treatment of breast cancer.

**Abstract:**

Backgrouds: Breast cancer remains a major global health challenge, with early diagnosis playing a crucial role in improving patient survival rates. Among the available diagnostic techniques, mammography is widely employed for early detection. However, its effectiveness is often constrained by the complexity of image interpretation, which makes automated detection methods increasingly vital. Methods: In this study, we propose an advanced approach that leverages 3D mammographic imaging and integrates Federated Learning (FL) to enable decentralized, privacy-preserving model training across multiple institutions. To evaluate the effectiveness of this approach, we assess various machine learning models, including Convolutional Neural Networks (CNNs), Transfer Learning architectures (VGG16, VGG19, ResNet50), and AutoEncoders (AEs), using 3D mammographic data. Results: Our results indicate that the CNN model achieves an accuracy of 97.30%, which improves slightly to 97.37% when the model is combined with Federated Learning, highlighting both the predictive performance and privacy-preserving advantages of our method. In contrast, Transfer Learning models and AutoEncoders exhibit lower accuracies that range from 48.83% to 89.24%, revealing their limitations in the context of this specific task. Conclusions: These findings underscore the effectiveness of the CNN-FL framework as a robust tool for breast cancer detection, showing that this approach offers a promising balance between diagnostic accuracy and data security—two critical factors in medical imaging.

## 1. Introduction

Breast cancer remains one of the leading causes of mortality among women worldwide, making early and accurate detection essential for improving survival rates. In recent years, deep learning has demonstrated remarkable capabilities in medical image analysis, particularly for breast cancer detection. However, training such models requires substantial amounts of data [1], and this poses significant challenges due to privacy concerns, data-ownership issues, and the inherently decentralized nature of medical data. Traditional centralized learning approaches, which aggregate data from multiple institutions into a single repository, have become increasingly impractical. They not only raise serious privacy risks but also often violate regulatory constraints, rendering large-scale data sharing infeasible.

To overcome these challenges, Federated Learning (FL) [2] has emerged as a promising alternative, enabling multiple institutions to collaboratively train machine learning models without exchanging sensitive patient data. In an FL framework [3], each institution trains a model locally on its own dataset and transmits only model updates—such as weight deltas or gradients—to a central server, which aggregates them to refine a global model. While FL has shown success in processing 2D medical images, its application to 3D imaging modalities [4], such as mammography and magnetic resonance imaging (MRI), remains underexplored. Although ~3D images provide richer diagnostic information, they also introduce additional computational and preprocessing complexities.

In this study, we propose a novel FL-based architecture specifically designed for the classification of 3D breast cancer images. Our approach effectively addresses the challenges associated with high-dimensional medical imaging by integrating advanced preprocessing techniques and optimized model architectures. To assess its performance, we conduct extensive experiments on a large-scale dataset of 3D breast images, demonstrating that our federated approach not only preserves patient privacy but also achieves accuracy comparable to that of centralized learning models.

The key contributions of this work are summarized as follows:

- We design a federated learning framework specifically tailored for classifying 3D breast cancer images, with a particular focus on Digital Breast Tomosynthesis.
- Our approach incorporates innovative preprocessing strategies and model optimizations to address the inherent challenges of 3D medical imaging.
- We validate our methodology using real-world clinical datasets, demonstrating its effectiveness and practical applicability in healthcare environments.

By leveraging federated learning, we enable the use of collaborative, privacy-preserving AI models that allow medical institutions to jointly advance breast cancer detection. This paradigm shift has the potential to enhance early diagnosis, improve patient outcomes, and foster significant progress in medical research [5].

The remainder of this paper is organized as follows: Section 2 reviews related work. Section 3 details our proposed approach. Section 4 presents the experimental results, and Section 5 discusses key findings. Finally, Section 6 concludes the paper and outlines future research directions.

## 2. Related Work

The prediction and classification of breast cancer have been central research topics for both medical professionals and data scientists. Numerous studies have explored the use of machine learning (ML) techniques based on feature extraction, as well as deep learning (DL) approaches, to improve early detection and prognosis. The existing research on this approach to breast cancer diagnosis can be categorized into two main groups: traditional machine learning methods and deep learning-based approaches.

Several traditional ML methods have been employed for breast cancer detection. In [6], El Naqa et al. applied Support Vector Machines (SVMs) to classify mammographic microcalcifications, contributing significantly to advances in breast cancer diagnosis. Another study proposed a breast cancer-detection system incorporating Principal Component Analysis (PCA), a Multilayer Perceptron (MLP), transfer learning, and an SVM [7]. The authors developed a new processing approach based on nine key variables and four fundamental machine learning techniques, achieving an accuracy of 86.97% on the Breast Cancer Coimbra dataset. However, integrating multiple methodologies often increases research complexity, leading to fragmented results.

In [8], a novel technique was introduced to enhance breast cancer detection in mammograms by leveraging wavelet transforms combined with neural networks. This approach, which was further refined through swarm intelligence techniques, significantly improved the accuracy of tumor identification. Such advancements in computer-aided detection systems have the potential to enable earlier and more reliable diagnoses.

Furthermore, an ensemble of classifiers—including Decision Trees, a Multilayer Perceptron Classifier (MLPClassifier), AdaBoost Classifier, and Gaussian Naive Bayes—was employed in [9] to predict breast cancer. This study utilized 30 extracted features—including standard error, mean, and worst values—from the widely used Wisconsin Breast Cancer Dataset (WBCD), providing valuable insights into various aspects of breast cancer analysis. Similarly, Singh et al. [10] conducted a comparative analysis of machine learning algorithms for breast cancer diagnosis and further proposed an autoencoder model for unsupervised breast cancer detection. Their objective was to identify the most relevant features associated with breast cancer using the Breast Cancer Wisconsin (Diagnostic) Dataset, which is available on Kaggle.

Despite significant advancements in the development of deep learning models for breast cancer classification, there has been only limited research on decentralized and privacy-preserving methodologies for breast cancer detection. To address this gap, this paper proposes a federated learning-based CNN model for breast cancer classification using 3D mammography data.

In [11], an effective deep learning model leveraging transfer learning was proposed for the automatic detection and diagnosis of breast cancer. The study utilized pre-trained CNN architectures—including InceptionV3, ResNet50, Visual Geometry Group Networks (VGG)-10, VGG-16, and~Inception-V2 ResNet—to extract features from the Mammographic Image Analysis Dataset (MIAS) [12]. The evaluation results demonstrated that the VGG16 model performed effectively in classification of mammographic images, excelling in accuracy, sensitivity, specificity, and other metrics.

The approach in [13] combined a CNN with an Unsupervised Extreme Learning Machine (US-ELM) to extract and cluster features from mammographic images. The CNN subdivided images into multiple regions for feature extraction at the subregion level, while the US-ELM clustered these features to detect tumor regions. However, the dataset used in this study included only about 400 subjects, limiting its generalizability and resulting in moderate accuracy.

In [14], a Deep Learning Assisted Efficient Adaboost Algorithm (DLA-EABA) was introduced for breast cancer detection. This study further employed CNN and LSTM architectures to analyze tumor characteristics for diagnostic purposes. The results demonstrated exceptionally high accuracy across different imaging modalities—including digital breast tomosynthesis, mammography, ultrasound, and MRI—with an overall accuracy of 97.2%.

Similarly, ref. [15] proposed a deep learning framework for breast cancer detection in mammograms. Their end-to-end training approach minimized the need for detailed lesion annotations, relying solely on image-level labels during the initial training phase.

Another approach was introduced in [16], where the authors proposed a Support-Valued Deep Neural Network (SNDN). Their methodology involved extracting entropy, geometric, and textural features from preprocessed images to assess the effectiveness of breast cancer-detection models.

Federated learning has also been explored in breast cancer research. In [17], Ma et al. developed a federated prediction model by integrating FL with CNN-based techniques. Their study demonstrated improved simulation performance across five types of cancer, achieving over 90% accuracy—outperforming single-model machines, tree models, linear models, and traditional neural networks. However, the study did not include comparisons with MLP-based models and did not address data-imbalance issues.

## 3. Methodology

**Figure 1:**
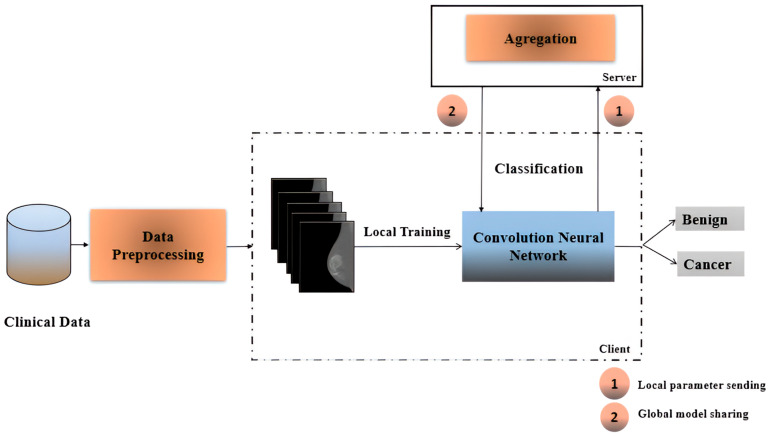
Workflow for global aggregation and local training.

This research presents a novel method for detecting breast cancer from 3D images using Federated Learning (FL). FL is a decentralized deep learning framework that enables multiple clients to collaboratively train a shared model without directly sharing sensitive data. With this technique, Convolutional Neural Networks (CNNs) can be trained on diverse image datasets collected from different hospitals and medical institutions while maintaining data privacy. No raw data are transmitted; instead, only model updates are sent to a central server, where they are aggregated to construct a global model. Each local model is trained on its respective dataset, ensuring that all data remain within institutional boundaries. The workflow illustrating the interaction between local and global models is shown in Figure 1. This collaborative approach enhances both the accuracy and generalization capabilities of the model while ensuring strict data confidentiality, making it particularly suitable for medical applications, where privacy is critical.

### 3.1. Data Preprocessing

**Figure 2:**
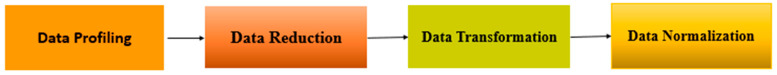
Steps of dataset preprocessing.

The preprocessing stage involves a sequence of transformations and refinements that are applied to the dataset to enhance its suitability for the model. These steps ensure that the data are well-structured and optimized for efficient processing. The entire procedure is illustrated in Figure 2. All 3D DICOM volumes were systematically preprocessed using the same method and projected along the *y*-axis to ensure uniformity across patients. We maintained dataset consistency by retaining the slices with the highest tissue contrast and diagnostic significance for selection. Using this approach, we obtained standardized 2D representations suitable for use in federated learning.

**Figure 3:**
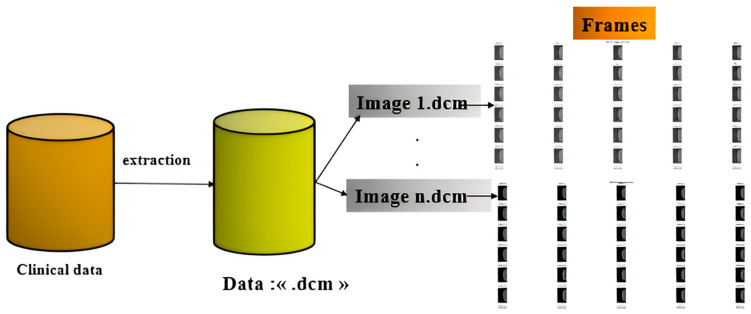
Transformation steps applied to breast cancer data.

- Data Profiling: The raw dataset is publicly available online and includes an extraction tool for retrieving MRI images of individual patients. Once obtained from the official repository, the dataset is imported into the working environment for analysis.
- Data Reduction: The initial database contains records from 5060 patients, categorized into four distinct classes: actionable, cancer, normal, and benign. To streamline the classification process, we extracted only the images relevant to a binary classification task, focusing on the “cancer” and “benign” categories.
- Data Transformation: To optimize model performance, a preprocessing step specifically designed for 3D MRI images was applied. Each DICOM (.dcm) image consists of multiple frames representing different slices or intensity levels. To extract these frames, the images were projected onto the *y*-axis using the values from the second channel. Additionally, visually distinct frames were selected for further analysis (cf. Figure 3). The dataset was then organized into separate training and testing subsets.
- Data Normalization: In this phase, image normalization was performed to eliminate intensity-related biases. Each image was first rescaled by dividing its pixel values by 255.0 to bring them into the [0, 1] range. Subsequently, intensity normalization was applied by dividing each image by its mean total intensity, ensuring consistent brightness levels across samples. This step prevents the model from learning exposure-related variations and allows it to focus on relevant structural patterns for classification.

#### 3.1.1. Overview of Federated Learning (FL)

Federated learning is an advanced machine learning paradigm that enables models to be trained across multiple decentralized devices or servers while ensuring that raw data remain local [18]. This approach is particularly valuable in contexts involving sensitive information, such as medical records, as it eliminates the need for direct data sharing. Instead of aggregating data in a central repository, federated learning gathers and integrates model updates from individual devices, thereby preserving data privacy. This methodology provides an effective balance between strong machine learning performance and strict data-confidentiality requirements [19].

**Figure 4:**
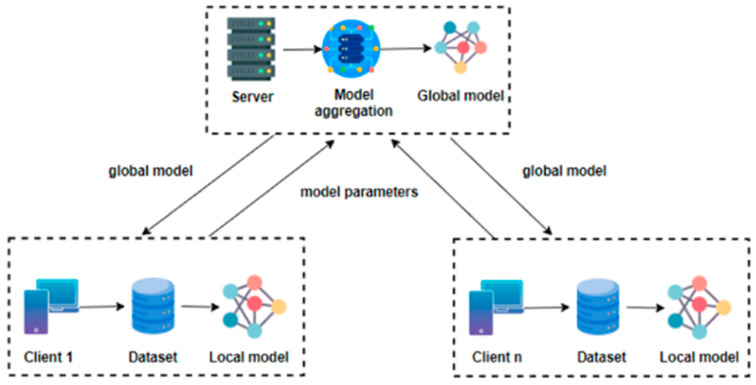
Decentralized Federated Learning architecture.

Federated learning consists of two main components: the server and the client. Additionally, Decentralized Federated Learning (DFL) techniques enable collaborative model training directly on individual devices, eliminating the need to transfer data to a centralized server for processing, as illustrated in Figure 4. No raw data are aggregate; instead, only model parameters or updates are exchanged, contributing to the improvement of the global model while preserving data privacy. This architecture effectively addresses privacy and security concerns by ensuring that clients communicate only model updates to the server and do not expose their datasets. The proposed workflow is presented in Figure 1, which outlines the steps performed on the client side while maintaining connectivity with the central server.

#### 3.1.2. Aggregation

The aggregation phase plays a fundamental role in federated learning, ensuring the enhancement of the global model by combining client-side updates. Each client trains its model independently using local data and transmits only the updated model weights or gradients to a central server.

This approach relies on the conventional Stochastic Gradient Descent (SGD), where clients update their local models before sending the computed updates to the server. The server then performs a weighted averaging process to refine the global model. The primary objective is to construct a robust global model by integrating diverse local knowledge [20]. By aggregating updates in this manner, the global model benefits from distributed and heterogeneous data while preserving user privacy. Furthermore, advanced aggregation strategies can account for variations in data distribution or computational capabilities among clients. This iterative process of aggregation and model refinement continues over multiple training rounds until the global model reaches the desired performance level [21].

We developed a federated learning system using the Flower framework. The parameter configuration for the aggregation function in our setup is provided in Table 1.

#### 3.1.3. Convolution Neural Network Model Used

Convolutional Neural Networks (CNNs) are widely used for visual classification tasks due to their ability to autonomously detect complex patterns in images. In this study, we employ a federated learning framework incorporating convolutional neural networks, with local models trained independently.

The designed CNN model consists of a structured sequence of convolutional layers that progressively extract meaningful and discriminative features from images. The architecture begins with 16 filters in the initial layer and scales up to 256 filters across five layers. Each convolutional layer is followed by a max-pooling operation, which reduces the spatial dimensions while retaining essential attributes. Finally, the extracted features are flattened and passed through two fully connected layers, culminating in a softmax layer that classifies the images as either benign or malignant cancer.

## 4. Experiment Results

This study aims to detect breast cancer cases in mammograms acquired from screening tests. To assess the effectiveness of the proposed methods, we carried out extensive experiments to identify the optimal configuration. Our approach was implemented using Google Colab, where we simulated a federated learning system with ten clients. Each client was responsible for defining model evaluation and training utilizing TensorFlow/Keras.

### 4.1. Collected Dataset

The dataset utilized in this study originates from breast cancer screening [22]. Digital Breast Tomosynthesis (DBT) is an advanced breast cancer screening technology approved in 2011 [23] that provides nearly 3D breast images; it is commonly referred to as 3D mammography. The DBT dataset comprises DICOM images categorized into normal, actionable, benign, and malignant cases (https://www.cancerimagingarchive.net/collection/breast-cancer-screening-dbt, accessed on 23 October 2025). To evaluate our proposed method, we extracted images belonging to two classes, cancer and benign, from the DBT DICOM files. Specifically, the cancer group contains 89 study files corresponding to 89 patients, whereas the benign category consists of 112 studies from 112 patients with benign masses.

**Figure 5:**
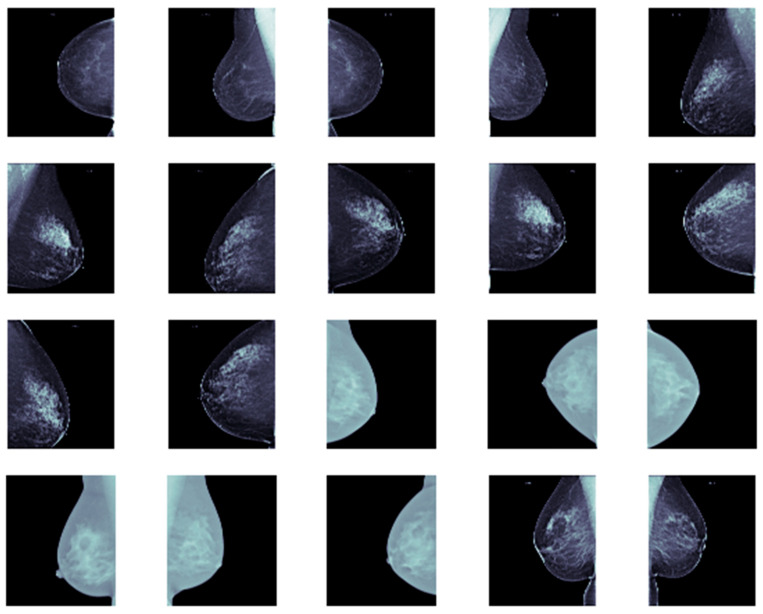
Example mammograms showing breast cancer.

Furthermore, we allocated approximately 80% of the dataset for training and validation, while the remaining 20% was reserved for testing. Each image was resized to 256 × 256. Representative samples from our dataset are shown in Figure 5.

To ensure a fair distribution of training data, we partitioned the dataset among multiple clients before initiating federated training. Each client received a specific data subset for local training. Proper data partitioning is essential in federated learning, as it enables clients to perform localized training while collaboratively enhancing the overall model.

### 4.2. Metric for Performance Evaluation

Several image-classification metrics have been utilized to evaluate the performance of the proposed method across all classes, including variance, F1-score, accuracy, precision, and recall [24]. Given its widespread use in breast cancer diagnosis, we selected classification accuracy as our primary metric (see Equation (1)). This metric quantifies the percentage of correctly identified test images and was used to assess our approach and compare it with existing methods.

(1) $$Accuracy=\frac{Number\;of\;correctly\;identified\;test\;samples}{Total\;number\;of\;test\;samples}$$

### 4.3. Experimental Settings

The experimental evaluation of the federated learning model applied to breast cancer classification on the DBT dataset follows a binary classification approach. The implementation was conducted using the Keras framework on top of TensorFlow. We leveraged the Flower federated learning framework to create and manage federated clients. Subsequently, we simulated a federated learning system utilizing a CNN model with 10 clients, where each client performed model training and evaluation using TensorFlow/Keras. The results demonstrated that federated learning enhances classification accuracy while maintaining patient data confidentiality.

Our decentralized model involves ten clients in total. In each training round, 50% of the clients are randomly selected to participate. Each client trains locally on its dataset for 50 epochs with a batch size of 32. The communication between clients and the server is conducted over 50 rounds. All breast images in the dataset were resized to 256 × 256 pixels. We used classification accuracy, a widely adopted metric in image classification, to assess our model. The hyperparameter settings for our federated learning architecture are summarized in Table 2.

**Figure 6:**
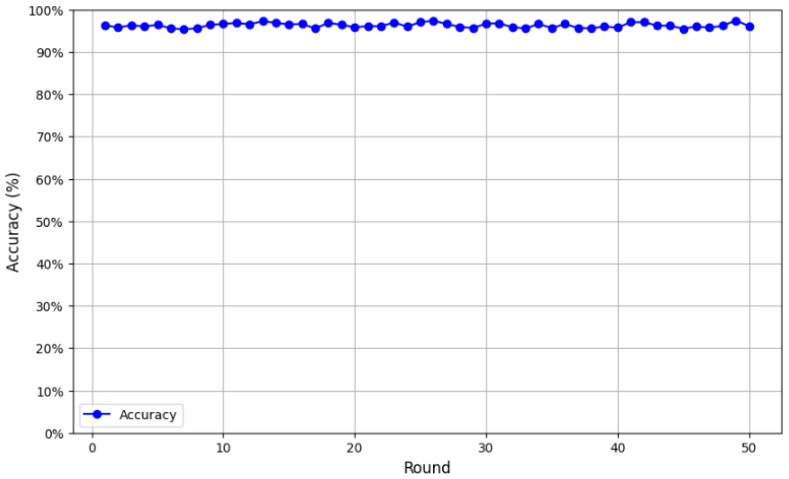
Accuracy improvement over validation rounds.

We first evaluated the CNN architecture with a single client training locally on its own data. On the test set, the model achieved an initial accuracy of 53%, which was relatively low. However, after the transition to federated learning, the model’s performance improved significantly. As illustrated in Figure 6, accuracy was monitored over 50 validation rounds, revealing a consistent upward trend. The model achieved an average accuracy of 95.27%, with performance peaking at 97.45% in the final round, confirming its robustness and efficiency.

**Figure 7:**
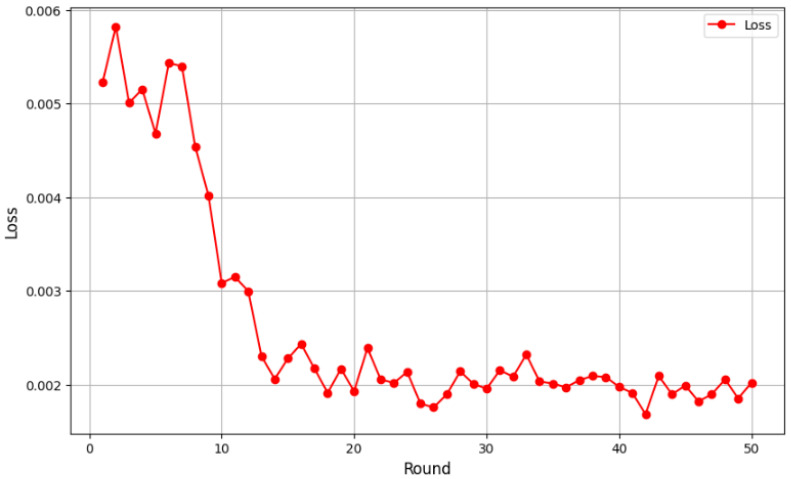
Loss reduction over validation rounds.

Following an initial testing phase, our federated model for breast cancer classification demonstrated outstanding performance, reaching an accuracy of 97.37%. Further validation even showed a slight improvement to an accuracy of 97.45% (Figure 7).

Initially, the loss values were relatively high, at around 0.005, as the global model had yet to effectively learn from the distributed data. However, from the fifth round onward, a clear downward trend emerged, indicating better adaptation to the aggregated data and improved feature extraction. Loss values continued to decline between rounds 10 and 20, eventually stabilizing below 0.003. By round 40, the loss ranged between 0.0019 and 0.002, suggesting model convergence. Minor fluctuations were observed, which is typical in federated learning due to data heterogeneity among clients, but they did not impact the overall downward trend, and the results indicated robust performance in 3D breast cancer image classification. To provide a thorough assessment of our models’ performance, we present evaluation metrics encompassing accuracy, sensitivity, specificity, precision and F1-score. Our FL + CNN model achieved an accuracy of 97.37%, with a sensitivity of 96.88% and a specificity of 97.50%. The high precision of 96.88% indicates that positive predictions are highly trustworthy, while the F1-score of 0.9688 confirms the balanced performance between precision and sensitivity (Figure 7).

A comparison of FL + CNN with the standalone CNN model reveals important insights. The standalone CNN achieved an accuracy of 97.30%, with a sensitivity of 96.25%, a specificity of 98.00%, a precision of 97.47%, and an F1-score of 0.9685. While both models demonstrate excellent performance, FL + CNN shows superior sensitivity (96.88% vs. 96.25%), detecting one additional cancer case compared to the standalone CNN. This improvement is clinically significant, as maximizing cancer detection is paramount in screening applications. The marginally lower specificity of FL + CNN (97.50% vs. 98.00%) represents an acceptable trade-off, resulting in one additional false positive that could be resolved through follow-up clinical examination.

Afterwards, the same dataset samples were later utilized for comparison with state-of-the-art classifiers. Table 3 presents performance comparisons of different classifiers using centralized data. We tested transfer learning models, including VGG19, VGG16 [25] and ResNet50 [26], which achieved 84.87%, 68.58%, and 48.83% accuracy, respectively. Additionally, we evaluated deep learning models such as CNN (https://www.tensorflow.org/tutorials/images/cnn?hl=fr, accessed on 23 October 2025) and Autoencoder (https://www.tensorflow.org/tutorials/generative/autoencoder?hl=fr, accessed on 23 October 2025), which yielded 97.30% and 89.24% accuracy, respectively. CNN outperformed the other models when applied to centralized data, achieving an accuracy of 97.30%.

Table 3 also compares our work with previous studies on breast cancer detection. We examined classifiers using centralized data, including deep learning and transfer learning approaches. As demonstrated, CNN-based models consistently outperformed other techniques, reinforcing the effectiveness of deep learning in extracting and classifying essential breast-image features. Our proposed method successfully harnesses the power of CNN in federated learning, further enhancing classification accuracy while ensuring data privacy.

## 5. Discussion

In this study, we employed 3D mammography DICOM images to classify breast cancer and assessed various machine learning and deep learning models, including convolutional neural networks (CNNs) and federated learning (FL) techniques.

We selected CNN as the baseline model primarily due to the significant preprocessing demands involved in extracting and structuring medical imaging data from archives, as well as the associated computational constraints. While more advanced 3D or hybrid CNN–Transformer architectures could potentially capture richer spatial features, their integration will be considered in future work once data-processing and hardware resources are optimized. The use of 3D mammography is crucial, as it provides a more detailed and comprehensive view of breast tissue, allowing more accurate tumor detection compared to traditional feature-extraction methods. As shown in Table 3, the CNN model effectively analyzed the complex 3D features of mammograms, achieving an accuracy of 97.30%. This outstanding performance is likely due to CNN’s ability to identify spatial hierarchies and complex patterns in image data.

However, the integration of Federated Learning (FL) with Convolutional Neural Networks (CNNs) resulted in an impressive accuracy of 97.37%. We chose to employ CNNs because they are highly effective for image-classification tasks, offering strong feature-extraction capabilities with relatively moderate computational demands. In contrast, fully 3D architectures, while powerful, require substantially greater computational resources.

Federated Learning enables decentralized model training across multiple devices or institutions, ensuring data privacy and security while still benefiting from the collective knowledge of the distributed datasets. This approach is particularly crucial in the field of medical imaging, where protecting patient data is of the utmost importance.

By combining CNNs with FL, our model not only achieves high accuracy, but also promotes collaborative learning among healthcare institutions without the need to share sensitive patient information, thus maintaining both performance and privacy. 

In comparison, the transfer learning models, such as VGG19, VGG16, and ResNet50, achieved lower accuracies that ranged from 48.83% to 84.38%. While these models benefit from pre-trained weights on large datasets, they still fall short when applied to 3D mammography, possibly due to their inability to effectively capture the features of the images. The AutoEncoder (AE), although unsupervised, yielded a strong result, with an accuracy of 89.24%, showing that unsupervised learning can still be effective for feature extraction from mammography images.

Overall, the results emphasize the effectiveness of combining CNN with Federated Learning in the context of 3D mammography. This approach not only delivers accurate results but also tackles privacy concerns, offering a promising solution for large-scale, collaborative breast cancer detection. Although 3D CNNs or hybrid volumetric methods could potentially preserve richer spatial context, their integration within federated frameworks remains computationally demanding and device-dependent. For this reason, we focused on 2D projections in this study. Future work will investigate volumetric CNNs and hybrid 2D/3D strategies to assess potential performance improvements. To ensure reproducibility, we assumed that the ten clients were equally dispersed in our experiments. Convergence and accuracy may be impacted by non-IID data, uneven client involvement, and disparate computing resources, all conditions that federated learning systems frequently encounter in practice. Only one dataset was used for the simulation in this study, which limits the evaluation scope. Additionally, a publicly available dataset such as that used here may not fully showcase the potential of the proposed method. Therefore, these points constitute limitations of the present study.

## 6. Conclusions

This study introduced a federated learning-based decentralized approach for breast cancer detection, eliminating the need for centralized data collection and preserving data privacy. Our methodology involved preprocessing mammographic images and leveraging a convolutional neural network (CNN) classifier within a federated learning framework to enhance classification accuracy. The implementation was conducted using Python (v 3.14.0), with the DBT dataset selected for experimental evaluation. Compared to existing techniques, our proposed model demonstrated superior performance, achieving an accuracy of 97.37%. This result highlights the model’s robustness and its potential for secure breast cancer detection without compromising sensitive patient data.

Despite these promising results, certain limitations remain. First, the study was conducted on a single dataset, which restricts the generalizability of the findings. Moreover, work that relies solely on publicly available datasets may not fully capture the complexity of real-world medical imaging data. Additionally, the proposed approach involves multiple computational steps, making it relatively time-intensive.

For future research, we plan to extend our work by testing the model on diverse breast cancer datasets to further validate its effectiveness. Enhancing image-preprocessing techniques and exploring more efficient federated optimization strategies could also contribute to improved performance and reduced computational overhead. By addressing these aspects, we aim to refine the proposed method, making it more adaptable for large-scale medical applications.

## Figures and Tables

**Table 1:** Configuration parameters for FedAvg with Flower.

Title 1	Title 2	Title 3
num-supernodes	Total number of clients	10
fraction-fit	Fraction of clients participating in training during each round.	1.0
fraction-evaluate	Half of the available clients will participate in the evaluation phase during each round	0.5
num-rounds	Number of rounds.	50
min-evaluate-clients	Ensures that at least five clients must participate in the evaluation phase for the round.	5

**Table 2:** Hyperparameter settings for federated learning architectures.

Hyperparameter	Configuration
Model	CNN
Number of clients	10
Aggregation method	FedAvg
Batch size	32
Number of rounds	50

**Table 3:** Performance comparison of various models on the preprocessed dataset.

Model	Technique	Accuracy
CNN	Deep Learning	97.30%
VGG19	Transfer Learning	84.38%
VGG16	Transfer Learning	68.58%
ResNet50	Transfer Learning	48.83%
AutoEncoder (AE)	Unsupervised Learning	89.24%
FL + CNN	Federated Learning	97.37%

## Data Availability

The datasets used and analyzed during the current study are publicly available at “The Cancer Imaging Archive” under the following link: https://www.cancerimagingarchive.net/collection/breast-cancer-screening-dbt/, accessed on 23 October 2025).
